# The c-Jun N-terminal kinase (JNK) pathway is activated in human interstitial cystitis (IC) and rat protamine sulfate induced cystitis

**DOI:** 10.1038/srep19670

**Published:** 2016-02-17

**Authors:** Jiang Zhao, Liang Wang, Xingyou Dong, Xiaoyan Hu, Long Zhou, Qina Liu, Bo Song, Qingjian Wu, Longkun Li

**Affiliations:** 1 Department of Urology, Second Affiliated Hospital, Third Military Medical University, Chongqing, 400037, China; 2 Departments of Urology, Cancer Institute/Cancer Hospital, Chongqing, 400030, China

## Abstract

The pathogenesis of bladder pain syndrome/interstitial cystitis (BPS/IC) is currently unclear. However, inflammation has been suggested to play an important role in BPS/IC. JNK downstream signaling plays an important role in numerous chronic inflammatory diseases. However, studies of the JNK pathway in BPS/IC are limited. In this study, we investigated the role of the JNK pathway in human BPS/IC and rat protamine sulfate (PS)-induced cystitis and examined the effect of the selective JNK inhibitor SP600125 on rat bladder cystitis. In our study, we demonstrated that the JNK signaling pathway was activated (the expression of JNK, c-Jun, p-JNK, p-c-Jun, IL-6 and TNF-α were significantly increasing in BPS/IC compared to the non-BPS/IC patients) and resulted in inflammation in human BPS/IC. Further animal models showed that the JNK pathway played an important role in the pathogenesis of cystitis. JNK inhibitors, SP600125, effectively inhibited the expression of p-JNK, p-c-Jun, IL-6 and TNF-α. The inhibition of these pathways had a protective effect on PS-induced rat cystitis by significantly decreasing histological score and mast cell count and improving bladder micturition function (micturition frequency significantly decreasing and bladder capacity significantly increasing). Therefore, JNK inhibition could be used as a potential treatment for BPS/IC.

Bladder pain syndrome/interstitial cystitis (BPS/IC) is a sterile bladder cystitis that is characterized by an increase in urinary frequency, urgency, pelvic pain, and other discomforts. In adult females in the United States BPS/IC symptoms are widespread and affect 3.3–7.9 million women^1^. Additionally, BPS/IC symptoms affect quality of life and social interactions^2^. The pathogenesis of BPS/IC is currently unclear. Many theories have been suggested to explain the pathogenesis of BPS/IC, such as epithelial damage, mast cell infiltration, autoimmunity, infection, and pelvic floor dysfunction^3^. However, inflammation has been suggested to have an important role of in both human and animal BPS/IC^4^,^5^,^6^.

Mitogen-activated protein kinases (MAPK) are a family of serine/threonine kinases that are evolutionarily conserved signal-transducing enzymes unique to eukaryotes. C-Jun N-terminal kinase (JNK) is a member of the MAPK superfamily and is an important signaling pathway involved in inflammation development. JNK is activated and phosphorylated in response to numerous stimuli (including oxidant stress and cytokines^7^,^8^. Subsequently, activated JNK phosphorylates c-Jun and contributes to the formation of the activator protein 1 (AP-1) transcription factor complex involved in the expression of many inflammatory genes^7^,^8^. Previous research suggests that JNK regulates the synthesis of many inflammatory cytokines (including IL-6 and TNF-α). JNK also responds to cytokines, such as TNF-α, IL-1 and growth factors^7^,^8^,^9^. Recent studies showed that JNK downstream signaling plays an important role in numerous inflammatory diseases, such as arthritis, colitis, systemic sclerosis and liver injury^8^,^9^,^10^,^11^,^12^,^13^. However, studies of the JNK pathway in BPS/IC are limited. In this study, we investigated the role of the JNK pathway in both human and animal BPS/IC and examined the effect of the selective JNK inhibitor SP600125 on rat bladder cystitis.

## Results

### Histological evaluations of human BPS/IC and PS-induced cystitis

**Figure 1:**
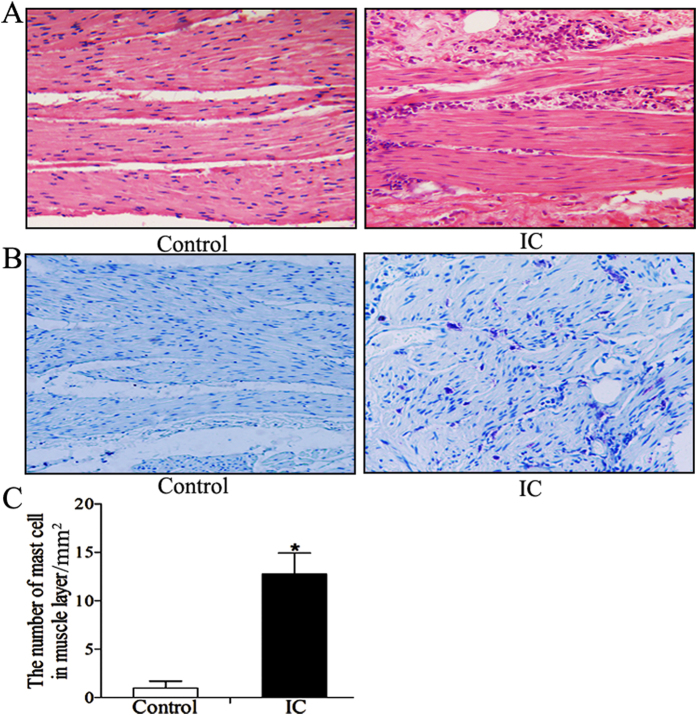
Histological evaluation in human BPS/IC. (**A,B**) Representative HE and toluidine blue staining (x20) photomicrograph images of numerous inflammatory cells and mast cell infiltration into the bladder muscular layer, arrows demonstrate inflammatory cells and mast cell. (**C**) The chart indicates the number of mast cells in muscular layer in control (n = 7) and BPS/IC humans (n = 6). The data are expressed as the mean ± SD, *P < 0.05, BPS/IC vs. control.

**Figure 2:**
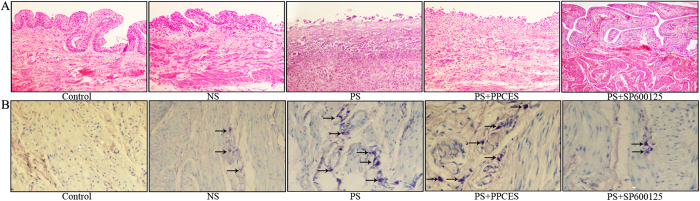
Histological evaluation in rat PS-induced cystitis. (**A,B**) Representative HE and toluidine blue staining (x20) photomicrograph images of pathologic changes and mast cell infiltration into the bladder muscular layer in PS-treated rats, arrows demonstrate mast cell.

In this study, bladder tissue from BPS/IC patients indicated thinning and edema in the epithelium with inflammatory infiltration in the lamina propria, as previously reported^14^,^15^. Compared with the control group, we found numerous mast cells (1.00 ± 0.71 vs 12.75 ± 2.18, p < 0.001, Fig. 1B,C) and inflammatory cells infiltrating the bladder muscular layer (Fig. 1A,B). HE (Fig. 2A) and toluidine blue (Fig. 2B) staining revealed severe epithelial damage, mucosal edema and inflammatory cell infiltration in the bladder wall of the PS-treated group (particularly mast cell) compared to the control group. However, the histological score and mast cells counts (Table 1) demonstrated that the inflammation was more severe in the PS and PPCES (PPCES vehicle containing 30% PEG-400/20% polypropylene glycol/15% Cremophor EL/5% ethanol/30% saline) + PS groups and more abate in the PS + SP600125 group.

### Expression of JNK, c-Jun, p-JNK, p-c-Jun, IL-6 and TNF-α in the human BPS/IC and rat PS-induced cystitis

**Figure 3:**
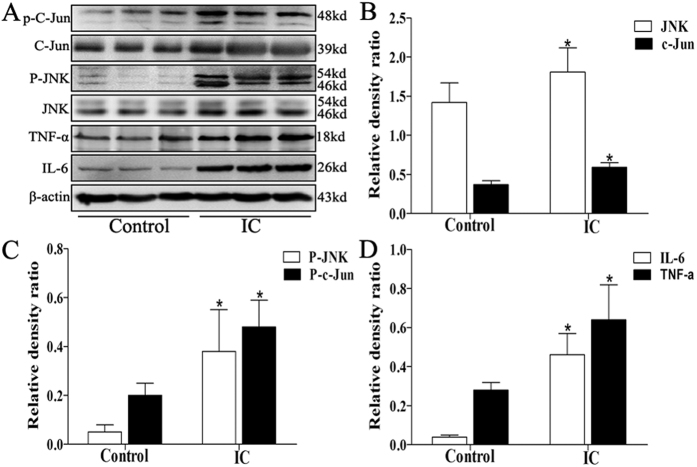
The expression of JNK, c-Jun, p-JNK, p-c-Jun, IL-6 and TNF-α changes in human BPS/IC using Western blot. (**A**) Typical protein bands showing the increased expression of JNK, c-Jun, p-JNK, p-c-Jun, IL-6 and TNF-α in BPS/IC bladder. (**B–D**) The chart shows the expression of JNK, c-Jun, p-JNK, p-c-Jun, IL-6 and TNF-α in control (n = 8) and BPS/IC humans (n = 9). The data are expressed as the mean ± SD, *P < 0.05, BPS/IC vs. control.

**Figure 4:**
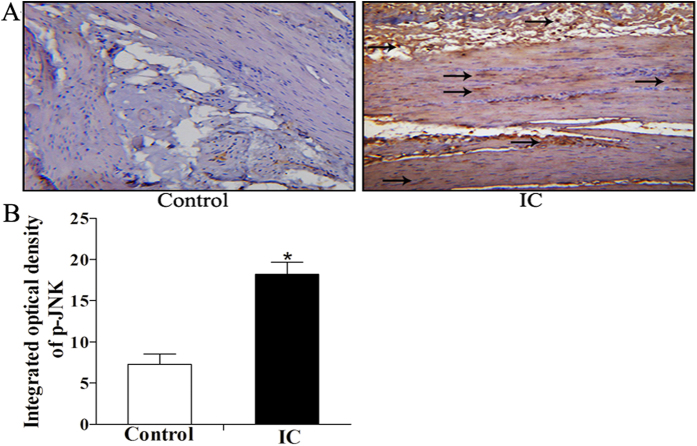
The expression and location of p-JNK changes in human BPS/IC using immunohistochemistry. (**A**) Representative immunohistochemistry (x20) shows p-JNK expression in the bladder muscles layers of mesenchymal cells (including inflammatory cells) and detrusor myocytes, arrows demonstrate the p-JNK expression. (**B**) The chart indicates the expression change of p-JNK in control (n = 7) and BPS/IC humans (n = 6). The data are expressed as the mean ± SD, *P < 0.05, BPS/IC vs. control.

**Figure 5:**
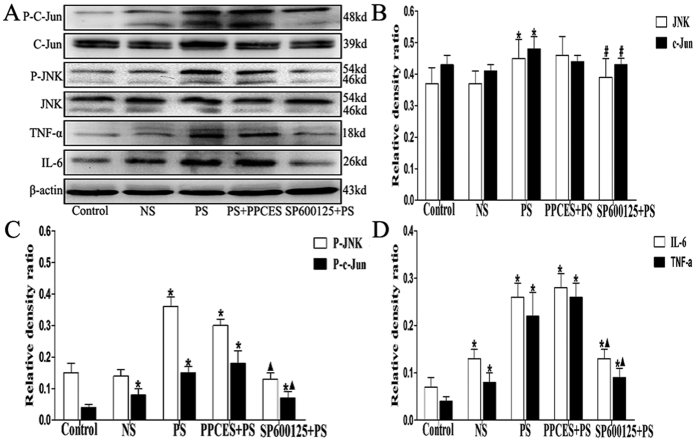
Evaluation of JNK, c-Jun, p-JNK, p-c-Jun, IL-6 and TNF-α changes in rat PS-induced cystitis using Western blot. (**A**) Typical protein bands showing increased expression of JNK, c-Jun p-JNK, p-c-Jun, IL-6 and TNF-α in PS-treated rats. (**B–D**) The chart shows the expression of JNK, c-Jun, p-JNK, p-c-Jun, IL-6 and TNF-α in PS-treated rats (N = 8). The data are expressed as the mean ± SD, *P < 0.05, NS, PS, PPCES + PS and SP600125 + PS vs. control, ^▲^P < 0.05, SP600125 + PS vs. PS and PPCES + PS, ^#^P < 0.05, SP600125 + PS vs. PS.

There were increases in JNK (1.42 ± 0.25 vs. 1.81 ± 0.31, P < 0.05), c-Jun (0.37 ± 0.05 vs. 0.59 ± 0.06, P < 0.05), p-JNK (0.05 ± 0.03 vs. 0.38 ± 0.17, P < 0.05), p-c-Jun (0.20 ± 0.05 vs. 0.48 ± 0.11, P < 0.05), IL-6 (0.04 ± 0.01 vs. 0.46 ± 0.11, P < 0.05) and TNF-α (0.28 ± 0.04 vs. 0.64 ± 0.18, P < 0.05) expression in BPS/IC bladders compared to control patients (Fig. 3A–D). As shown in Fig. 4, p-JNK was found in the bladder muscle layers of mesenchymal cells and detrusor myocytes and were increases in BPS/IC bladders(18.19 ± 1.47 vs. 7.92 ± 1.25, P < 0.05). In the PS-treated rats the expression of p-JNK, p-c-Jun, IL-6 and TNF-α (Fig. 5A,C,D) was increased compared with the control group (P < 0.05). The signaling changes were more obvious in the PS and PPCES + PS groups and were less apparent in the PS + SP600125 group. Additionally, in the PS + SP600125 group, the expression of p-JNK, p-c-Jun, IL-6 and TNF-α (Fig. 5A,C,D) were significantly decreased compared with the PS and PPCES + PS groups (P < 0.05).

### The changes of urodynamic parameters in the PS-treated rats

**Figure 6:**
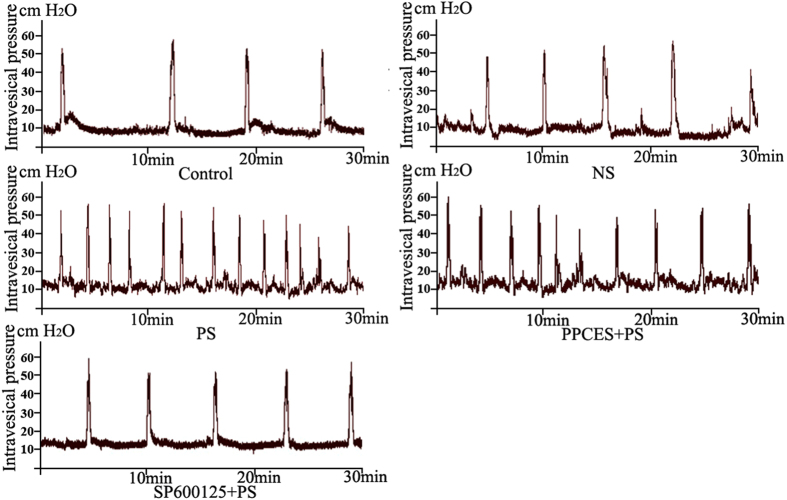
Representative cystometric traces of control, NS, PS, PPCES + PS and SP600125 + PS groups.

As shown in Table 2 and Fig. 6, compared to the control group, the maximum pressure (MF) was significantly increased (P < 0.05) and the intercontraction interval (ICI) and bladder capacity (BC) were decreased (P < 0.05) in the NS (normal saline), PS and PPCES + PS groups. However, compared to the PS and PPCES + PS groups the MF was significantly decreased (P < 0.05) and the ICI and BC were significantly increased (P < 0.05) in the SP600125 + PS group. There was no statistical significance found in basal pressure (BP) and MP parameters in the NS, PS, PPCES + PS and SP600125 + PS groups.

## Discussion

BPS/IC is still somewhat difficult to treat. Although there are many theories, the etiology of BPS/IC remains unknown^6^,^16^,^17^. Recent studies have focused on urothelial damage to illustrate the pathogenesis BPS/IC and the results suggested that the epithelial injury and repair abnormalities will lead to urine toxic substances (such as) resulting in a ‘leaky’ epithelium. which resulted in a cascade of reactions, causing symptoms, tissue injury and disease progression with the result secondary inflammation^17^,^18^. Furthermore, many studies have found the presence of mast cell infiltration and neurogenic inflammation in BPS/IC^6^,^16^,^19^,^20^. These findings suggest that the bladder wall and inflammation play irreplaceable roles in the etiology of BPS/IC. However, there is limited research examining the bladder submucosal tissue-detrusor layer and the regulatory changes that occur in the BPS/IC muscle layer.

It is well established that inflammatory disease is characterized by increased expression of multiple inflammation-related proteins that frequently result in irreparable pathology. During inflammation development, JNK is activated and phosphorylated. Phosphorylated JNK then phosphorylates c-Jun, which is a component of the transcription factor AP-1 complex. In complex with other DNA binding proteins, AP-1 regulates the transcription of numerous inflammation-related protein genes (including cytokines, immunoglobulins and inflammatory enzymes, etc.) involved in inflammation^7^,^8^,^9^. In many inflammatory diseases, including arthritis, colitis, fibrosis and liver injury, JNK and its downstream proteins are activated. Additionally, blocking JNK activation can significantly improve the inflammatory response and protect tissue and organ damage^7^,^8^,^9^,^12^,^21^. In this study, we found a significant activation of JNK signaling (p-JNK and p-c-jun, Figs 3 and 4) in the bladder muscle layer in IC/BPS patients. The JNK pathway can be activated by various stimuli, including oxidative stress and inflammatory mediators (such as IL-6 and TNF-α)^9^,^22^,^23^. In this study, we used western blotting (Fig. 3A,D) to demonstrate IL-6 and TNF-α expression were increased in the bladder muscle layer in BPS/IC patients. In addition, HE (Fig. 1A) and toluidine blue (Fig. 1B) staining confirmed the presence of numerous inflammatory cells and mast cell infiltration into the bladder muscular layer^19^,^20^. In a BPS/IC bladder, IL-6 and TNF-α can be secreted by a variety of immune cells (including monocytes, macrophages, mast cells) and bladder detrusor myocytes^19^,^20^,^24^,^25^. Additionally, the overexpression of IL-6 and TNF-α can promote mast cell recruitment, proliferation and maturation and can lead to bladder structural damage and apoptosis of the urinary epithelium^17^,^19^,^24^,^25^,^26^. In summary, we hypothesize that epithelial damage causes toxic substances in urine to infiltrate the bladder muscular layer. These changes cause inflammatory cell and mast cell release of the inflammatory mediators (such as IL-6 and TNF-α) in the bladder muscular layer. Subsequently, the overexpression of inflammatory mediators caused JNK pathway activation which further causes the release of inflammatory mediators and mast cell infiltration. Finally, the deterioration of the microenvironment forms a cycle that results in persistent inflammation and epithelial damage.

Previous studies have demonstrated that protamine sulfate (PS) damages the bladder glycosaminoglycan (GAG) layer. Once the GAG layer is damaged, the bladder mucosa would results in a loss of the normal permeability barrier and the toxic factors would be transferred from urine to the bladder interstitium and would cause to the inflammatory reactions, which was the secondary to the leak of toxic factors (such as potassium) through a defective GAG layer^15^,^17^. Thus, the animal model of PS-induced cystitis was extensively used for studies on inflammation changes in BPS/IC^15^,^17^,^27^. The results of our animal experiments are consistent with previous reports showing significant inflammation after PS treatment (Fig. 2 and Table 1) using histological analysis^15^,^27^. Additionally, we demonstrate that JNK signaling pathway related proteins (p-JNK and p-c-jun, Fig. 5A,C) and the expression of inflammatory cytokines (IL-6 and TNF-a, Fig. 5A,D) are significantly increased in the PS and PPCES + PS groups. Interestingly, we found that mast cell infiltration in the bladder muscle layer is also significantly enhanced in the PS and PPCES + PS groups. These results are consistent with human BPS/IC outcomes and support our hypothesis.

SP600125 is a small molecule reversible ATP-competitive inhibitor of protein kinases that inhibits targets all of the three different isoforms of JNK. SP600125 treatment significantly reduces inflammation by effectively blocking the JNK pathway and showed a marked protective effect in colitis, arthritis and liver injury^7^,^8^,^9^,^21^. In this study, we demonstrated that the JNK signaling pathway related proteins (p-JNK and p-c-jun, Fig. 5A,C) and inflammatory cytokines (IL-6 and TNF-a, Fig. 5A,D) are significantly decreased in the SP600125 + PS group. In addition to the reduced inflammation, we also found that the inflammation and mast cell infiltration into the bladder muscular layer was significantly reduced after SP600125 treatment. Previous studies suggest that reducing the expression of inflammatory cytokines (IL-6 and TNF-a) can improve bladder voiding function^14^,^15^,^27^,^28^. In our urodynamic studiesv(Fig. 6 and Table 2), we found that the bladder contraction frequency increased in the PS and PPCES + PS groups. Furthermore, voiding intervals shortened and the bladder volume decreased. However, this trend of bladder function damage is significantly recovered in the SP600125 + PS group. This finding suggests a better recovery of micturition function due to the SP600125 mediated reduction of inflammation caused by JNK pathway inhibition. These results confirmed that SP600125 effectively inhibited the phosphorylation of c-jun and reduced the expression of IL-6 and TNF-a, which protected PS-treated rats.

In conclusion, these studies demonstrate that the JNK signaling pathway was activated and leads to inflammation in BPS/IC. Further animal models showed that the JNK pathway plays an important role in the pathogenesis of cystitis. JNK inhibitors, SP600125, effectively inhibit the expression of p-JNK, p-c-Jun, IL-6 and TNF-α. The inhibition of these pathways has a protective effect on PS-induced rat cystitis by significantly decreasing inflammation and improving bladder function. Therefore, JNK inhibition could be used as a potential treatment for IC.

## Materials and Methods

### Human bladder specimen harvest and classification

Six bladder specimens from female BPS/IC patients (age: 34–64 years old) were provided via cystectasy or cystectomy by the Department of Urology, Second Affiliated Hospital to Third Military Medical University according to the NIDDK diagnosis criteria and were classified as the BPS/IC group. Another Seven bladder specimens from patients receiving a radical cystectomy with bladder cancer were classified as the control group. All patients provided informed consent. This study was approved by the institutional review board of Second Affiliated Hospital to Third Military Medical University and was carried out in accordance with established national and institutional ethical guidelines regarding the involvement of human subjects and the use of human tissues for research.

### Animal model establishment with protamine sulfate-induced cystitis

This study was approved by the Research Council and Animal Care and Use Committee of the Third Military Medical University, China (approval no. SYXK20070002) and was carried out in accordance with relevant guidelines and regulations regarding the involvement of animal subjects and the use of animal tissues for research. In this study, 80 female SD rats weighing 200–230 g were randomized into control, saline (NS), protamine sulfate (PS), PPCES + PS, and SP600125 + PPCES + PS (sp600125 + PS) groups. There were 16 rats in each group. In the NS group, a PE-50 transurethral catheter was inserted into the bladder to empty the bladder, and then, 0.5 mL of saline was infused intravesically and remained for 30 min. In the PS group, the bladders were pretreated with 0.5 mL of PS (30 mg/mL, sigma, P3369) by intravesical infusion, as previously described^14^,^15^. In the PPCES + PS and SP600125 + PS groups the rats were intraperitoneally injected with SP600125 in the PPCES vehicle (30% PEG-400/20% polypropylene glycol/15% Cremophor EL/5% ethanol/30% saline) two hours after the intravesical infusion of PS. The final volume was 5 ml/kg^9^,^29^. All rats were treated three times a week for 1 month and the bladders were harvested within 48 h after the last treatment.

### Histological study and expression of JNK pathway with western blot analysis in BPS/IC and PS -induced cystitis

The rat bladders were harvested and then divided longitudinally into two individual sections for histological study and western blots. One bladder section was fixed in 10% buffered formalin, and the other sections were frozen at −80 °C. To determine inflammatory changes, both bladder specimens from patients (n = 13) and each group of rats (n = 8) were embedded in paraffin and cut into 5 μm sections. The sections were then stained with hematoxylin–eosin (H.E.)and toluidine blue (Shengong Biotech, TE847). For each tissue section, 5 random fields at 20× magnification were examined. A histological score was calculated and the number of mast cells was counted. The tissue section analyses were evaluated by a blinded pathologist. The bladder histological score was assessed using a previously described four-point scoring system^14^,^15^. The protocol for staining patient specimens for p-JNK immunohistochemistry has been previously described^12^,^14^. The following antibodies were used: rabbit polyclonal anti-Phospho-JNK antibody (1:200, CST #9164) and horseradish peroxidase-conjugated goat anti-rabbit IgG (1:300 Zhongshan Inc, ZB-2301). The expression of huaman p-JNK was used The Pro-Plus Image 6 software analysis, the integrated optical density (IOD) of p-JNK was recorded. All of the bladder specimens from patients (n = 13) and rats (n = 8 per group) were used for western blotting as previously reported^30^. The primary antibodies included the following: rabbit polyclonal anti- Phospho-JNK antibody (1:1000, CST, #9251), rabbit polyclonal anti- Phospho-c-jun antibody (1:800, CST #9164), a rabbit polyclonal anti-c-jun antibody (1:600, Santa Cruz Biotechnology, sc-44), a rabbit polyclonal anti-jnk antibody (1:100, Abcam, ab10664), rabbit polyclonal anti- IL6 antibody (1:1000, abcom, ab6672), rabbit polyclonal anti-TNF-α antibody (1:800, CST, #3707), rabbit polyclonal anti-TNF-α antibody (1:800, GeneTex, GTX74120), mouse polyclonal anti-β-actin (1:1000, Santa Cruz Biotechnology, sc-47778), horseradish peroxidase-conjugated goat anti-mouse IgG (1:3000, Zhongshan Inc, ZB2305) and goat anti-rabbit IgG (1:1500 Zhongshan Inc, ZB-2301). The protein band images were collected and the relative optical density (R.O.D) by calibrating agianst the β-actin as internal control was analyzed with molecular image, ChemiDoc XRS + Image System (Bio-Rad Laboratories, Hercules, CA, USA).

### Detrusor overactivity determination with bladder cystometry

Cystometry was performed as described previously^15^,^31^,^32^. The rats were anesthetized with urethane (1.2 g/kg, subcutaneously, sigma, U2500). A PE 50 catheter was inserted into the bladder for saline infusion at body temperature (37–38 °C) at a rate of 8 ml/h, and the intravesical pressure was continuously recorded. After stabilization for 30 minutes, the urodynamic parameters, including basal pressure (BP), maximum pressure (MP), micturition frequency (MF), intercontraction interval (ICI), and bladder capacity (BC), were recorded.

### Statistical analysis

All data are presented as the mean ± standard deviation. The statistical analysis was performed using SPSS 16.0 by a blinded investigator. The inflammatory data were evaluated using the Kruskal-Wallis test. The other data were analyzed with one-way ANOVA followed by the Bonferroni test for multiple comparisons. P < 0.05 is considered statistically significant

## Additional Information

**How to cite this article**: Zhao, J. *et al.* The c-Jun N-terminal kinase (JNK) pathway is activated in human interstitial cystitis (IC) and rat protamine sulfate induced cystitis. *Sci. Rep.* **6**, 19670; doi: 10.1038/srep19670 (2016).

## Figures and Tables

**Table 1:** Histological evaluation in rat PS-induced cystitis (n = 8).

Group	Normal	NS	PS	PPCES + PS	SP600125 + PS
Histological score	0.25 ± 0.46	0.87 ± 0.64^a^	2.87 ± 0.35^a^	2.62 ± 0.52^a^	1.00 ± 0.7^a^,^b^
Mastocytes number/mm2	0.52 ± 0.50	1.02 ± 0.83	7.45 ± 1.82^a^	8.07 ± 1.73^a^	2.07 ± 0.92^a^,^b^

Data are expressed as the mean ± SD, ^a^P < 0.05, NS, PS, PPCES + PS and SP600125 + PS vs. control, ^b^P < 0.05, SP600125 + PS vs. PS and PPCES + PS.

**Table 2:** Cystometric parameters changes in rat PS-induced cystitis (n = 8).

Group	Normal	NS	PS	PPCES + PS	SP600125 + PS
MP(cmH2O)	54.25 ± 6.73	55.75 ± 5.60	58.50 ± 6.35	57.62 ± 6.14	57.62 ± 4.07
MF(No/h)	6.87 ± 0.83	10.25 ± 1.49^a^	21.25 ± 1.75^a^	20.87 ± 2.03^a^	9.00 ± 1.31^b^
BC (ml)	1.18 ± 0.14	0.80 ± 0.11^a^	0.38 ± 0.03^a^	0.38 ± 0.03^a^	0.91 ± 0.13^a^,^b^
ICI (min)	8.83 ± 1.06	5.96 ± 0.86^a^	2.84 ± 0.24^a^	2.90 ± 0.28^a^	6.80 ± 1.02^b^

BP: basal pressure; MP: maximum pressure; MF: micturition frequency, ICI: intercontraction interval; BC: bladder capacity. Data are expressed as the mean ± SD, ^a^P < 0.05, NS, PS, PPCES + PS and SP600125 + PS vs. control, ^b^P < 0.05, SP600125 + PS vs. PS and PPCES + PS.
